# Data for the generation of RNA spatiotemporal distributions and interpretation of Chk1 and SLBP protein depletion phenotypes during *Drosophila* embryogenesis

**DOI:** 10.1016/j.dib.2017.05.008

**Published:** 2017-05-13

**Authors:** Fabio Alexis Lefebvre, Louis Philip Benoit Bouvrette, Julie Bergalet, Eric Lécuyer

**Affiliations:** a Institut de Recherches Cliniques de Montréal (IRCM), Montréal, Québec, Canada; b Département de Biochimie, Université de Montréal, Montréal, Québec, Canada; c Division of Experimental Medicine, McGill University, Montréal, Québec, Canada

## Abstract

The data presented in this article is related to the research article entitled “Biochemical Fractionation of Time-Resolved *Drosophila* Embryos Reveals Similar Transcriptomic Alterations in Replication Checkpoint and Histone mRNA Processing Mutants” (Lefebvre et al., 2017) [1]. This article provides a spatiotemporal transcriptomic analysis of early embryogenesis and shows that mutations in the checkpoint factor *grapes*/Chk1 and the histone mRNA processing factor SLBP selectively impair zygotic gene expression. Here, lists of transcripts enriched in early syncytial embryos, late blastoderm embryos, cytoplasmic and nuclear extracts of blastoderm embryos are made public, along with transcription factor motif occurrence for genes enriched in each context. In addition, extensive lists of genes down-regulated upon Chk1 and SLBP protein depletion in embryos are released to enable further analyses.

## **Specifications Table**

**Table:** 

Subject area	Biology
More specific subject area	Embryogenesis, Transcriptomics, Histone mRNA processing
Type of data	Table, images (microscopy, correlation heatmaps),
How data was acquired	RNA-seq (Illumina Hi-Seq. 2000), Fluorescence microscopy (Leica DM5500B)
Data format	Analyzed
Experimental factors	Time after egg laying and subcellular localization
Experimental features	*Drosophila wt* embryos were collected and flash-frozen 0–45 min after egg laying (min AEL) and 90–180 min AEL. Chk1 (*grp*^*fs1*^) and SLBP (*Slbp*^*10/12*^ and *Slbp*^*10/15*^) mutant embryos were collected and frozen 0–180 min AEL.
Data source location	Montreal, QC, Canada
Data accessibility	Raw (fastq files) and processed (.xls) RNA-seq data was deposited at GEO with accession number GSE89001

## **Value of the data**

• Lists of transcripts enriched in early syncytial and in late blastoderm *wt* embryos can be useful to study zygotic genome activation in *Drosophila.*
• Lists of transcripts enriched in nuclear and cytoplasmic extracts of blastoderm *wt* embryos can be useful to study the dynamics of nucleocytoplasmic shuttling and the determinants of RNA localization.
• Lists of transcripts down-regulated in *grp* and *Slbp* mutant embryos can be useful to study replication stress and histone depletion response in embryogenesis.

## 1 Data

We used staged embryo collections, biochemical fractionation and RNA-seq to define transcripts enriched in early vs late embryogenesis and in the nuclear vs cytoplasmic compartments. We then analyzed mutants of DNA replication checkpoint and histone mRNA processing factors. We identified over 2500 transcripts with compromised levels in these mutants, most of which are normally enriched in late nuclei [1].

## 2 Experimental design, materials and methods

### 2.1 Time-resolved embryo collections

We sought to establish repertoires of transcripts enriched in ‘*Early/E*’ cleavage cycles (0–45 min AEL, mitotic cycles 1–6) and in ‘*Late/L*’ blastoderm (90–180 min AEL; mitotic cycles 10–14) embryos. *Wt* 90–180 min AEL embryos were collected for 90 min and kept at 25 °C for 90 more minutes. Embryos were collected and dechorionated as previously described [2]. We used formaldehyde fixation and DAPI staining [3] to validate the staged collections by fluorescence microscopy on a Leica DM5500B microscope (Fig. S1). We found that early samples all adopted preblastoderm syncytial morphology, while late samples were all cellularized blastoderm embryos.

### 2.2 RNA extraction and sequencing

In addition to time-resolved analysis, we aimed to assess the spatial distribution of transcripts during embryogenesis. We derived ‘*Nuclear/N*’ and ‘*Cytoplasmic/C*’ extracts from 90 to 180 min AEL blastoderm embryos through a subcellular fractionation protocol [4]. We extracted RNA of total early embryos and cytoplasmic and nuclear extracts of late embryos as previously described [4]. We used TRIZol^™^ according to the manufacturer׳s instructions and then purified RNA samples using the RNA Clean & Concentrator^™^-5 system (Zymo). We performed on-column DNAse I treatment (New England BioLabs); washes and elution were as described by the manufacturer. Samples with high RNA integrity number (R.I.N.) were selected to prepare RNA-seq libraries upon ribosomal RNA depletion using the RiboMinus kit (Thermo Fisher). Sequencing was performed using the Illumina HiSeq. 2000 machine and TruSeq PE Clusterkit v3-cBot-HS. FastQC v0.10.1 was used to assess read quality. Tophat v2.0.10 was used for alignment on the *Drosophila melanogaster* BDGP5.78/dm^3^ genome. Normalized read counts were derived from BAM files with Deseq. 2 and a threshold of 5 reads was applied in downstream analyses.

### 2.3 Identification of transcripts with asymmetric spatiotemporal expression

To define transcripts enriched in the early, late, nuclear and cytoplasmic material, we calculated enrichment ratios based on normalized read counts. For example, a nuclear enrichment score *N*_y_ for a given transcript was defined as *N*_y_=2×*N*_x_/(*E*_x_+*C*_x_+*N*_x_), where *E*_x_, *C*_x_ and *N_x_* correspond to normalized read counts mapped in the early syncytial, cytoplasmic blastoderm and nuclear blastoderm libraries. Gene lists were sorted based on this metric, yielding exponential distributions. We aimed to define an unbiased cut-off reflecting the dispersion of each exponential function and selected values exceeding *y*_min_×(*y*_max_/*y*_min_)^0.6^ for each distribution [5], [6]. This approach lead to the selection of genes responsible for the top 40% dispersion of the enrichment score distribution. Further methodological information on the selection approach is provided in the research article. We defined 2821 nuclear transcripts, 1187 cytosolic transcripts, 364 early syncytial and 360 late blastoderm transcripts (Table S1). Nuclear and late blastoderm groups showed extensive overlap (Table S2). DAVID [7] was used to retrieve enriched GO terms and associated statistical significance values from protein-coding gene selections, with all *Drosophila* proteins as a background. Gene ontology enrichment analysis [7] showed that nuclear and late blastoderm selections were largely functionally redundant (Table S3). We used previous surveys of zygotic genes established by De Renzis et al. [8] and Lécuyer et al. [9], which revealed sizeable overlap with transcripts enriched in the nuclear fraction of blastoderm embryos (Tables S4 and S5). We retrieved 2000 bp sequences upstream of the transcription start for genes enriched in each spatiotemporal context and used HOMER [10] “known motif” resource to identify overrepresented transcription factor binding motifs with the default settings. We provide detailed lists of motif occurrence for genes enriched in each context (Tables S6–S9). Binding sites of the Caudal transcription factor were predominant in the promoter regions of transcripts expressed in early embryos (Table S6), while Zelda binding sites were overrepresented among transcripts enriched in late blastoderm embryos (Table S7) and corresponding nuclear extracts (Table S8). The DNA replication element (DRE) was enriched among transcripts found in cytoplasmic extracts of blastoderm embryos (Table S9).

### 4.4 Impact of *grapes*/Chk1 and SLBP mutations

We then aimed to contrast the impact of Chk1 and SLBP protein depletions on early embryogenesis. We collected 0–180 min AEL embryos from mothers with the allelic combinations *grp* ^*fs1*^, *Slbp*^*10/12*^ and *Slbp*^*10/15*^. Mutant embryos were obtained through crosses of trans-heterozygous mothers with *wt* OregonR males. We also collected *wt* embryos using the corresponding time-line, proceeded to RNA-seq and identified genes displaying a 4-fold or greater decrease in the mutants based on normalized read counts (Table S10). The functional signatures of transcripts affected in the three mutants were related to transcription factor activity (Table S11). We conducted HOMER analysis of promoter sequences for genes exhibiting a 4-fold or greater decrease in each mutant and provide detailed lists of motif occurrence in each case (Tables S12–14). TATA-box were most prevalent for all three mutants. In addition, a strong majority of transcripts compromised in the mutants were enriched in nuclear extracts of *wt* blastoderm embryos (Fig. S2). When comparing transcript levels in *wt* and mutant samples, we found that Spearman׳s (ρ) and Pearson׳s (r) coefficients were weak for the population of transcripts enriched in late blastoderm embryos (Fig. S3). To extend this analysis, we independently compared the fold change resulting from each mutation considering six groups of asymmetrically expressed transcripts. Levels of ovarian transcripts (Table S15), transcripts enriched in early syncyctial embryos (Table S16) and transcripts enriched in the cytoplasmic extracts of blastoderm embryos (Table S17) were not severely compromised by the mutations. By contrast, levels of transcripts defined as ‘purely zygotic’ [8] by De Renzis et al. (Table S18), transcripts enriched in late blastoderm embryos (Table S19) and transcripts enriched in nuclear extracts of blastoderm embryos (Table S20) were severely down-regulated in *grp* ^*fs1*^, *Slbp*^*10/12*^ and *Slbp*^*10/15*^ mutants.

## Appendix A Supplementary material

Fig. S1. Morphological validation of staged embryo collections. *Wild-type* embryos collected 0–45 min AEL (left) and 90–180 min AEL (right) were dechorionated, fixed, stained with DAPI and examined to determine corresponding mitotic cycle.

Fig. S2. Spatiotemporal distribution of transcripts impaired in *grp*^fs1^, *Slbp*^10/12^ and *Slbp*^10/15^ embryos. Venn diagrams of genes exhibing a 4-fold or greater decrease in *grp*^fs1^ (top), *Slbp*^10/12^ (center) and *Slbp*^10/15^ (bottom) and groups of RNAs enriched in early syncytial (E), late blastoderm (L), cytoplasmic (C) and nuclear (N) blastoderm extracts.

Fig. S3. Correlation heatmaps of spatiotemporally-restricted transcript levels in *wt*, *grp*^fs1^, *Slbp*^10/12^ and *Slbp*^10/15^ embryos. Correlation heatmaps of normalized read count distributions in *wt*, *grp*^fs1^, *Slbp*^10/12^ and *Slbp*^10/15^ embryo extracts. Correlations were independently derived for groups of RNAs enriched in each spatiotemporal group. Spearman׳s (*ρ*) and Pearson׳s (*r*) coefficients are indicated with associated *P*-values designated by asterisks. Color intensity is proportional to *r*.
