# Supplementary figures and images for: Data for the generation of RNA spatiotemporal distributions and interpretation of Chk1 and SLBP protein depletion phenotypes during Drosophila embryogenesis
Source: Data Brief. 2017 May 13;13:28–31. doi: 10.1016/j.dib.2017.05.008 (PMC5440278; doi:10.1016/j.dib.2017.05.008)

**0-45 minutes AEL**

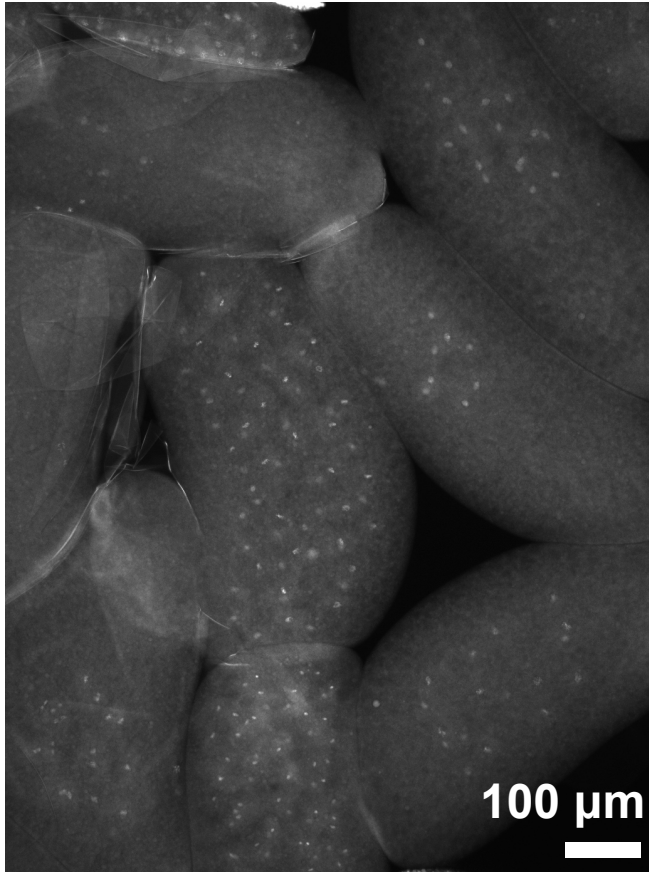

**Mitotic cycles 2-9**

**90-180 minutes AEL**

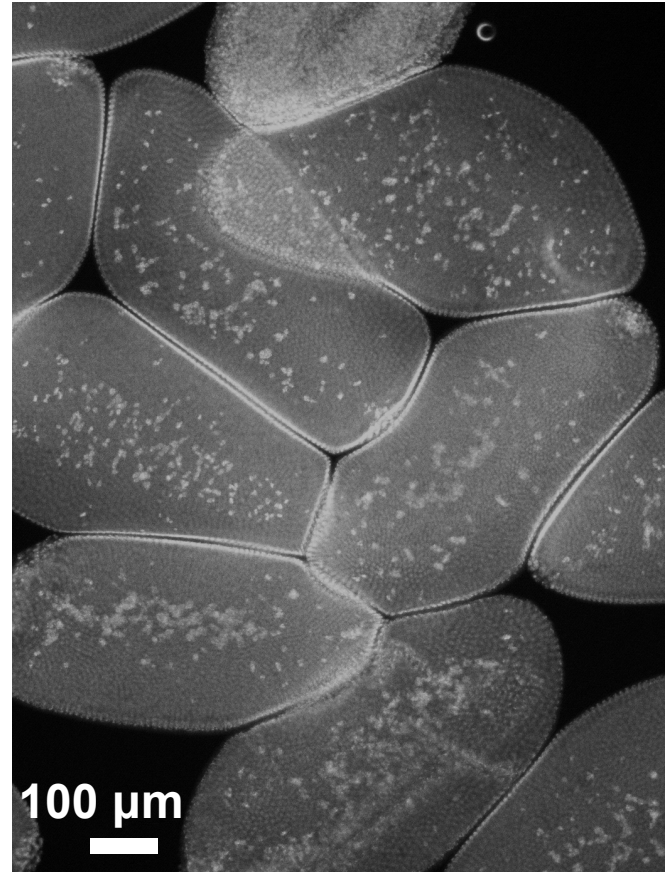

**Mitotic cycles 10-13**

Supplement: Supplementary file 3 — Fig. S1. Morphological validation of staged embryo collections. Wild-type embryos collected 0–45 min AEL (left) and 90–180 min AEL (right) were dechorionated, fixed, stained with DAPI and examined to determine corresponding mitotic cycle. [file mmc3.pdf]

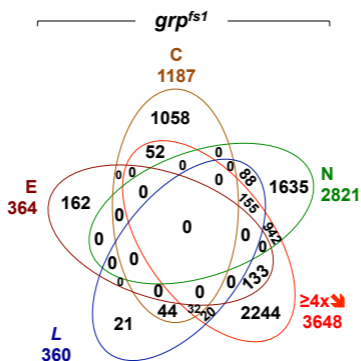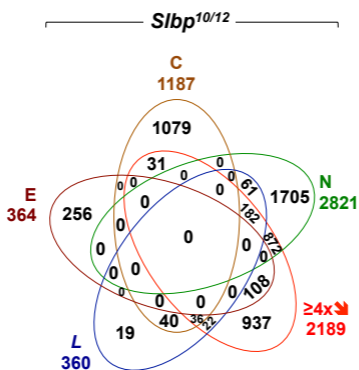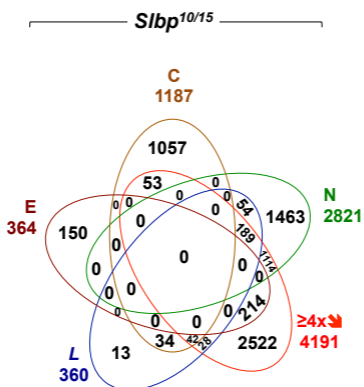

Supplement: Supplementary file 4 — Fig. S2. Spatiotemporal distribution of transcripts impaired in grpfs1, Slbp10/12 and Slbp10/15 embryos. Venn diagrams of genes exhibing a 4-fold or greater decrease in grpfs1 (top), Slbp10/12 (center) and Slbp10/15 (bottom) and groups of RNAs enriched in early syncytial (E), late blastoderm (L), cytoplasmic (C) and nuclear (N) blastoderm extracts. [file mmc4.pdf]

### early syncytial

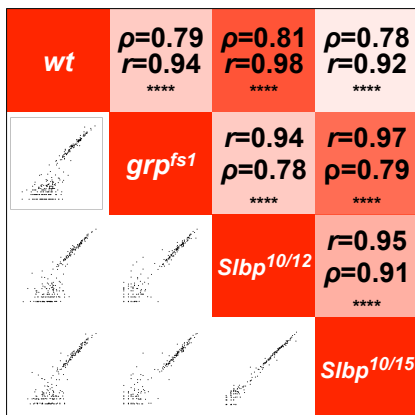

### late blastoderm

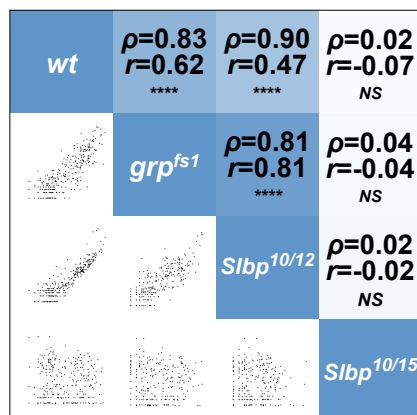

### late cytoplasm

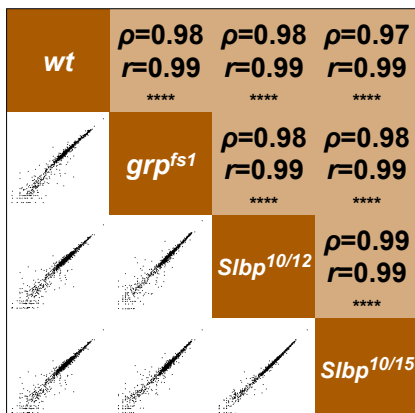

### late nuclear

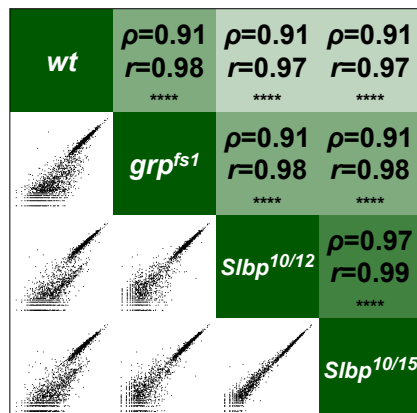

Supplement: Supplementary file 5 — Fig. S3. Correlation heatmaps of spatiotemporally-restricted transcript levels in wt, grpfs1, Slbp10/12 and Slbp10/15 embryos. Correlation heatmaps of normalized read count distributions in wt, grpfs1, Slbp10/12 and Slbp10/15 embryo extracts. Correlations were independently derived for groups of RNAs enriched in each spatiotemporal group. Spearman׳s (ρ) and Pearson׳s (r) coefficients are indicated with associated P-values designated by asterisks. Color intensity is proportional to r. [file mmc5.pdf]
